# Antiedematogenic Evaluation of *Copaifera langsdorffii* Leaves Hydroethanolic Extract and Its Major Compounds

**DOI:** 10.1155/2015/913152

**Published:** 2015-05-21

**Authors:** Ricardo Andrade Furtado, Cristiane Teixeira Vilhena Bernardes, Mauro Nogueira da Silva, Karina Furlani Zoccal, Lúcia Helena Faccioli, Jairo Kenupp Bastos

**Affiliations:** School of Pharmaceutical Sciences of Ribeirão Preto, University of São Paulo, 14040-903 Ribeirão Preto, SP, Brazil

## Abstract

Inflammatory disorders affect many people worldwide, and medicinal plants are used to ameliorate these health problems. This paper reports the antiedematogenic and analgesic evaluation of *Copaifera langsdorffii* Desf. leaves hydroethanolic extract (Cop) and two of its isolated compounds: quercetin-3-*O*-*α*-l-rhamnopyranosyl (quercitrin) and kaempferol-3-*O*-*α*-l-rhamnopyranosyl (afzelin). For that, the following experimental protocols were undertaken locomotor performance, writhing induced by acetic acid, antinociceptivity induced by formalin, hot plate latency, paw oedema induced by carrageenan and dextran, and cell migration induced by lipopolysaccharide (LPS), as well as the measurement of nitric oxide (NO), tumor necrosis factor alpha (TNF-*α*), interleukin 6 (IL-6), and interleukin 10 (IL-10) in macrophages. Neither the extract nor the isolated compounds displayed analgesic activity. The obtained results showed that *C. langsdorffii* extract possesses antiedematogenic properties acting on peripheral sites, whereas quercitrin and afzelin are not involved. Moreover, these properties are not associated with cell migration inhibition, TNF-*α*, IL-6, or IL-10 regulation.

## 1. Introduction

*Copaifera langsdorffii* Desf. (Leguminosae-Caesalpinioideae) is popularly known as “copaiba.” It is a large tree that grows widely in Brazil, especially in Amazonas, Pará, and Ceará States [1]. There are previous published works using the hydroethanolic extract of* C. langsdorffii* leaves (Cop) for urolithiasis in rats [2], nephrolithiasis in rats [3], genotoxicity in mice [4], and carcinogenicity in rats [5].

The inflammatory processes are the body's physiological responses to different stimulus, such as mechanical traumas and infections. Natural products have showed an important role in the treatment of inflammatory diseases [6] and* C. langsdorffii* has high medicinal and economic potential for the development of new herbal medicine, given the pharmacological activities already described. Taking this information into account, this paper reports the antiedematogenic and analgesic evaluation of the Cop and two of its compounds: quercetin-3-*O*-*α*-l-rhamnopyranosyl (quercitrin) and kaempferol-3-*O*-*α*-l-rhamnopyranosyl (afzelin).

## 2. Material and Methods

### 2.1. Plant Material, Extract Preparation, and Isolation

Leaves of* C. langsdorffii* were collected in the Campus of the University of São Paulo, Ribeirão Preto, SP, Brazil. The plant material was identified by Dr. Milton Groppo, a Botanist Professor of the University of São Paulo, and a voucher specimen (SPFR 10120) has been deposited in the herbarium of the* Faculdade de Filosofia, Ciências e Letras*, Ribeirão Preto, São Paulo, Brazil. Dried leaves were grounded and exhaustively extracted with ethanol : water 7 : 3 solution. The lyophilized extract was suspended in CH_3_OH : H_2_O 9 : 1 and partitioned successively with hexanes, CH_2_Cl_2_, and ethyl acetate (EtOAc).

Chromatographic analyses were undertaken in an HPLC Shimadzu SCL-10AVP (Kyoto, Japan) multisolvent delivery system equipped with a (Shimadzu SPD-M10Avp) photodiode array detector. Analyses were performed using the analytical reverse phase column C_18_ CLC-ODS (M) 25 cm × 4.6 mm (Shimadzu) with a particle diameter of 5 *μ*m. The mobile phase consisted of a mixture of increasing proportions of CH_3_OH in water with trifluoroacetic acid 0.01% (TFA). The elution program was 15–50% within 45 min, followed by 50–90% within 65 min and 90–15% within 70 min.

The EtOAc fraction (2.5 g) was chromatographed on HSCCC (high-speedy countercurrent chromatography). The chromatography was performed using interconnected 2 mm diameter columns of 102 mL and 105 mL with rotation of 850 rpm. The solvent ratio was hexanes :* n*-BuOH 1 : 1 (mobile phase) and CH_3_OH : H_2_O_2_ 0.4 : 1 (stationary phase), which exhibited settling time of 25 s. The values of partition coefficients were calculated from the data of the area of individual peaks in the chromatograms of the mobile and stationary phases of the solvent system. The mobile phase was eluted at the head-tail direction. Ten-milliliter fractions were combined after TLC analysis. Two fractions were purified in a preparative HPLC SPD-20 A UV-DAD with software* LC-Solution Single* (C_18_ Prep-ODS; 20 mm × 25 cm; Shimadzu) using an elution program consisting of 35–80% CH_3_OH in water (v/v) (20 min).

The nuclear magnetic resonance spectra (NMR) ^1^H, ^13^C NMR, and spectrometric dimensional techniques were recorded on spectrometer (Bruker-Avance DRX500), operating at 500 MHz (^1^H-NMR) and 125 MHz (^13^C-NMR). The samples were prepared in Aldrich deuterated dimethyl sulfoxide (DMSO-d_6_).

### 2.2. Animals

Male Wistar rats (130–180 g) and Swiss mice (20–30 g) were provided by the Central Animal House of University of São Paulo, Ribeirão Preto. Animals were housed in 12 h light-dark cycles at 22 ± 1°C with free access to food and water. The experiments were carried out in accordance with the guidelines for the care of laboratory animals [7]. It was approved by the Ethical Committee for Animal Care of the University of São Paulo (process numbers 09.1.1373.53.6 and 11.1.471.53.7). Saline vehicle (control; 0.9%), Cop, quercitrin, and afzelin were administered by gavage (10 mL/kg) and the number of animals was the minimum necessary to show consistent results.

### 2.3. Locomotor Performance and Toxicity Evaluation

Groups of four Swiss mice were observed during the first four hours to signs of general toxicity, restlessness, lethargy, aggressiveness, breathing, salivation, tearing, extremities cyanosis, piloerection, and mortality. In the 15th day the mice were euthanized, followed by necropsy and macroscopic observation of the organs. The method followed the requirements of OECD 423 [8]. The treatment groups were saline vehicle, Cop (30, 100, 300, and 1000 mg/kg), quercitrin (3, 10, 30, and 100 mg/kg), and afzelin (3, 10, 30, and 100 mg/kg). For evaluating locomotor performance a plastic box measuring 45 × 45 × 20 cm was used, with the bottom divided into nine equal areas (15 × 15 cm). The number of areas crossed by four paws of the animals was counted during six minutes and taken as an index behavior.

### 2.4. Acetic Acid-Induced Writhing Response

Mice were randomly assigned to groups with six mice. The saline vehicle, indomethacin (10 mg/kg, Sigma-Aldrich, batch 115K0689), Cop (100, 200, and 400 mg/kg), quercitrin (100 mg/kg), and afzelin (100 mg/kg) were administered orally by gavage (10 mL/kg) 60 min before intraperitoneal injection of 0.6% v/v acetic acid at 10 mL/kg. The writhing response was measured during 20 min after injection of acetic acid and expressed as writhing numbers [9].

### 2.5. Formalin Test

Mice were randomly assigned in groups of six. Twenty microliters of 2.5% formalin was injected into the dorsal surface of the right hind paw 60 min after oral administration by gavage of saline vehicle, indomethacin (10 mg/kg), and Cop (100, 200, and 400 mg/kg). Morphine (2.5 mg/kg, i.p., Pasmodex, batch 29386101) was administered 30 min before formalin injection. Then, mice were observed for 30 min after the injection of formalin, and the amount of time spent licking the injected hind paw was recorded [10].

### 2.6. Hot Plate Test

Groups of six mice were placed on the heated surface (55 ± 1°C; DS37 Ugo Basile, Italy) and latency between the placement and responses of shaking, licking of the paws, or jumping was recorded. A 20 s cutoff was used to prevent tissue damage. Measurements were taken at 0, 30, 60, and 90 min after treatment. The treatment groups received the doses of 100, 200, and 400 mg/kg of Cop and 4 mg/kg, i.p. of morphine. The percentage of maximal possible effect (MPE%) was calculated as follows: MPE% = (postdrug latency − basal latency)/(cutoff time − basal latency) × 100% [11].

### 2.7. Carrageenan and Dextran-Induced Rat Paw Oedema

Groups of six rats received injections of 0.1 mL of *λ*-carrageenan (100 *μ*g/paw, Sigma-Aldrich, batch 116K0105) or dextran (60 *μ*g/paw, Sigma-Aldrich, batch 091M13302V) into the right hind paw. In the left paw was injected equal volume of saline solution (0.9%). The treatment groups were control, indomethacin (10 mg/kg) or dexamethasone (1 mg/kg), and Cop (100, 200, and 400 mg/kg). The samples were administered 60 minutes before injection of carrageenan or dextran and the paw volume was measured for five hours at one-hour intervals for carrageenan and for two hours at 30 min intervals for dextran in a plethysmometer (insight EFF 304, Insight, Brazil). The inhibition of edema was calculated by the volume difference between the paws of each animal, compared with the control [12].

### 2.8. Cell Migration Assay

Groups of six rats were intraperitoneally injected with 100 ng/cavity of lipopolysaccharide (LPS; Mants,* Escherichia coli* serotype 0101: B4, batch 2630) 1 h after the administration of Cop (100, 200, and 400 mg/kg) or dexamethasone (1 mg/kg). After 4 h, animals were euthanized and the abdominal cavity was washed with 10 mL of PBS. The exudates were transferred to plastic tubes and total number of cells was determined in Neubauer chamber. For differential count, the supernatant was discarded after centrifugation (400 g for 10 min at 10°C), and the pellet was resuspended in 300 *μ*L of 3% albumin (Nutricell). The cells were fixed in glass slides, stained using the method of Rosenfeld, and 100 cells were analyzed by optical microscopy for counting the number of mononuclear cells, neutrophils, and eosinophils [13].

### 2.9. Peritoneal Macrophages Activation and Experimental Design

The resident peritoneal macrophages were obtained by injection of cold phosphate buffer solution (PBS) 1x (5 mL, 3x) into euthanized mice abdominal cavity. The peritoneal fluid was centrifuged (400 g for 10 min at 10°C). The cells were resuspended in RPMI 1640 medium supplemented with 10 mM L-glutamine, 100 U/mL penicillin, 100 U/mL streptomycin, and 10% fetal bovine serum (RPMI-c; Gibco, batch 210100K). The viability was confirmed by the Trypan blue dye exclusion technique. Cells were plated in 96-well culture plates (Zellkultur testplatte 96F, 92096, Switzerland) at a density of 2 × 10^5^ cells/well in RPMI medium. Two hours later, the plates were washed with PBS and the medium was replaced with fresh medium to remove nonadherent cells. The cells were incubated for 18 h in RPMI-c in an incubator containing a moisture atmosphere and 5% of CO_2_ at 37°C [14].

Cop (30, 60, and 120 *μ*g/mL) was added two hours after cells either prestimulated or not with LPS (0.4 *μ*g/mL). Cells incubated only with dimethyl sulfoxide (DMSO) 0.02% were used as control. After 24 h of incubation, culture supernatants were harvested and stored in a freezer at −20°C. NO and cytokine production were evaluated in supernatants by Griess method and ELISA, respectively. Cytotoxicity, NO, and cytokine production of cell were determined as described below.

### 2.10. Cytotoxicity Assay

Cytotoxicity was evaluated using the 3-(4,5-dimethylthiazol-2-yl)-2,5-diphenyltetrazolium bromide (MTT) colorimetric assay (Sigma-Aldrich) [15]. The cells were incubated with Cop at different concentrations associated or not with LPS for 24 h. The supernatants were removed and the cells were incubated with 5% MTT in RPMI-c for 4 h. Then, 150 *μ*L of DMSO was added to each well and maintained at room temperature until complete solubilization of the precipitated. Absorbance was measured at 570 nm with a spectrophotometer (mQuanti, Bio-Tek Instruments, Inc., Winooski, VT) and was directly proportional to cell viability.

### 2.11. NO Production

The amount of nitrite (NO_2_^−^) present in the supernatants was measured as an indicator of NO production using the Griess method [16]. The amount of NO_2_^−^ in the supernatants was calculated using a standard curve with serial NaNO_2_ dilutions. The assay was performed in quadruplicate, and the absorbance at 540 nm was recorded 10 min after addition of NaNO_2_.

### 2.12. Cytokine Production

The concentrations of tumor necrosis factor-*α* (TNF-*α*) and interleukins (IL) 6 and 10 in culture supernatants of macrophages were quantified by ELISA using specific antibodies (purified and biotinylated) and cytokine standards, according to manufacturer's instructions (R&D Systems, Minneapolis, USA). The optical densities were measured at 405 nm. Cytokine concentrations were determined using a standard curve established with the appropriate recombinant cytokine (expressed in pg/mL).

### 2.13. Statistical Analyses

The results are presented as the mean ± SEM. The statistical significance among the groups was assessed using the one-way analysis of variance (ANOVA), followed by Tukey's test. *P* values less than 0.05 were considered an indication of significance, as compared to the control group.

## 3. Results

### 3.1. Phytochemical Analysis

**Figure 1:**
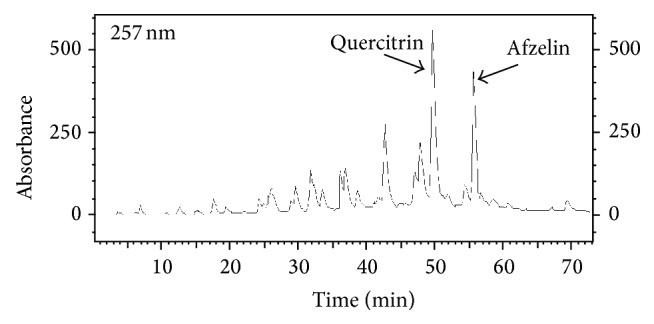
Chromatogram obtained in HPLC-UV-DAD of Cop with wavelength of 257 nm. C_18_ column (4.6 × 250 mm, 5 mM), mobile phase: (A) water and 0.01% trifluoroacetic acid and (B) MeOH, gradient elution: 15–50% of B in 45 min, 50–90% of B by 65 min, and 90–15% of B by 70 minutes followed by 2 minutes of 15% B; flow rate: 1 mL/min.

The crude extract yielded 33.9%, and the partition fractionation with organic solvents yielded the following crude fractions: hexanes (13%), CH_2_Cl_2_ (12%), EtOAc (27%), and water (48%), respectively. HPLC analysis showed that the major compounds in the crude extract were afzelin and quercitrin (Figure 1). On HSCCC, the partition coefficients were 0.6 to quercitrin and 0.95 to afzelin. The retention of the stationary phase before sample injection was 85%. The TLC analysis furnished fractions F_8–14_ (320 mg), F_15–23_ (215 mg), F_24–26_ (65 mg), F_27–33_ (93 mg), F_34–41_ (267 mg), and F_42–51_ (570 mg). Among the HSCCC obtained fractions, F_15–23_ and F_34–41_ displayed purities of approximately 90%, and, after purification in HPLC, purities were above 95% for both quercitrin (108.9 mg) and afzelin (94.5 mg).

All compounds were characterized by ^1^H- ^13^C-NMR and spectrometric dimensional obtained data, in comparison with literature [17], and only ^1^H-NMR data are showed below:

  Quercetin-3-*O*-*α*-l-rhamnopyranosyl (quercitrin; PubChem CID 5280459) displayed ^1^H-NMR *δ* (DMSO-*d*_6_) chemical shifts of 6.20 [1; s], 6.39 [1; s], 7.30 [1; s], 6.86 [1; d; *J* = 8.3], 7.25 [1; d; *J* = 8.3], 5.25 [1; s], 3.97 [1; s], 3.51 [1; dd; *J* = 9.1, 2.6], 3.15 [1; m], 3.20 [1; m], and 0.81 [3; d; *J* = 6.1].
  Kaempferol-3-*O*-*α*-l-rhamnopyranosyl (afzelin; PubChem CID 5316673) displayed ^1^H-NMR *δ* (DMSO-*d*_6_) data similar to quercitrin, but with major differences at ring B for 7.74 [2; d; *J* = 8.8] and 6.91 [2; d; *J* = 8.8] to afzelin and 7.30 [1; s], 6.86 [1; d; *J* = 8.3], and 7.25 [1; d; *J* = 8.3] to quercitrin.

### 3.2. Evaluation of Locomotor Performance and Toxicity

Both crude extract and isolated compounds did not cause significant behavioral and physiological changes in the animals within the tested doses (data not shown).

### 3.3. Effects on Models of Nociception

**Figure 2:**
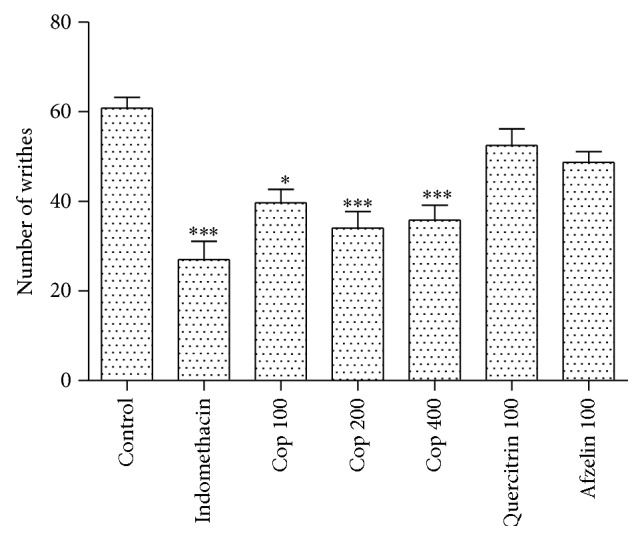
Cop (100, 200, and 400 mg/kg), quercitrin (100 mg/kg), and afzelin (100 mg/kg) effects on acetic acid-induced abdominal constriction in mice. Each bar represents the mean ± SEM. Symbols above the bars denote statistical difference in comparison with the control group (^∗^ *P* < 0.05 and ^∗∗∗^ *P* < 0.001).

A significant reduction in writhing of 40.3% was observed for the group treated with 200 mg/kg of Cop. On the other hand, the compounds quercitrin and afzelin did not display significant activity at a dose of 100 mg/kg (Figure 2).

**Figure 3:**
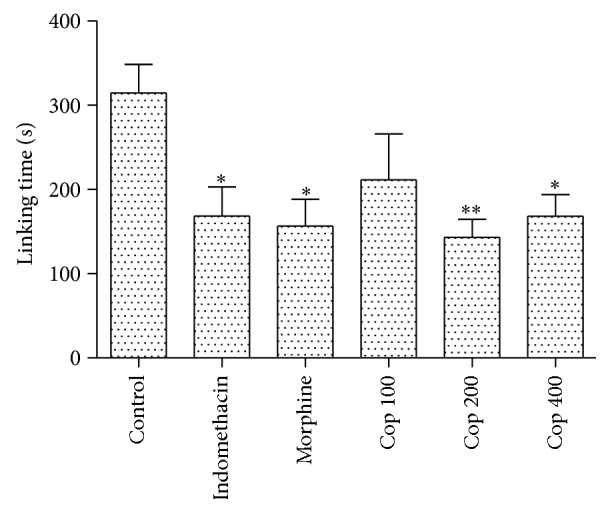
Cop (100, 200, and 400 mg/kg) effects on phase II of the nociceptive behavior induced by intraplantar injection of formalin. Each bar represents the mean ± SEM. Symbols above the bars denote statistical difference in comparison with control (^∗^ *P* < 0.05, and ^∗∗^ *P* < 0.01).

The results depicted in Figure 3 show that Cop (200 and 400 mg/kg) significantly inhibited the nociception in phase II of formalin-induced licking with maximal inhibition of 54.5% but did not change phase I (data not shown). Moreover, the absence of analgesic activity was confirmed by the administration of Cop, which did not produce any significant increase in latency in the hot-plate test (data not shown).

### 3.4. Evaluation of Antiedematogenic Properties

**Figure 4:**
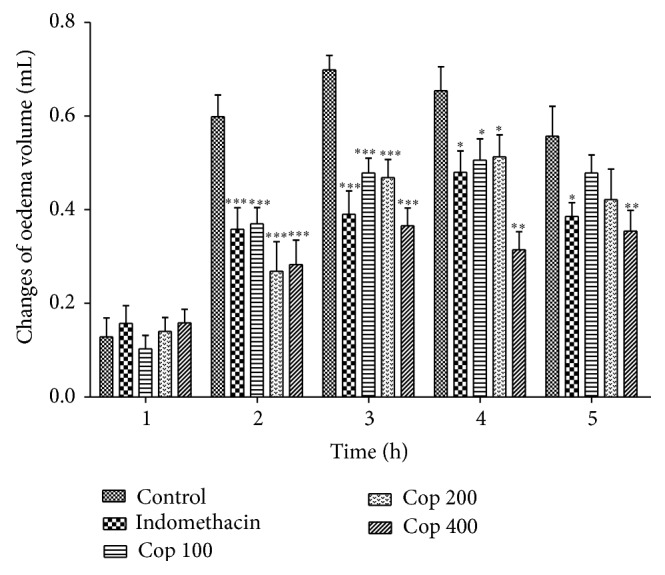
Effect of* C. langsdorffii* extract (Cop) and indomethacin treatments on carrageenan-induced rat paw oedema. Results are shown as mean oedema volume ± SEM. Time presented in hours at 1, 2, 3, 4, and 5 hours after carrageenan treatment. Statistical difference in comparison with the control group (^∗^ *P* < 0.05, ^∗∗^ *P* < 0.01, and ^∗∗∗^ *P* < 0.001).

**Figure 5:**
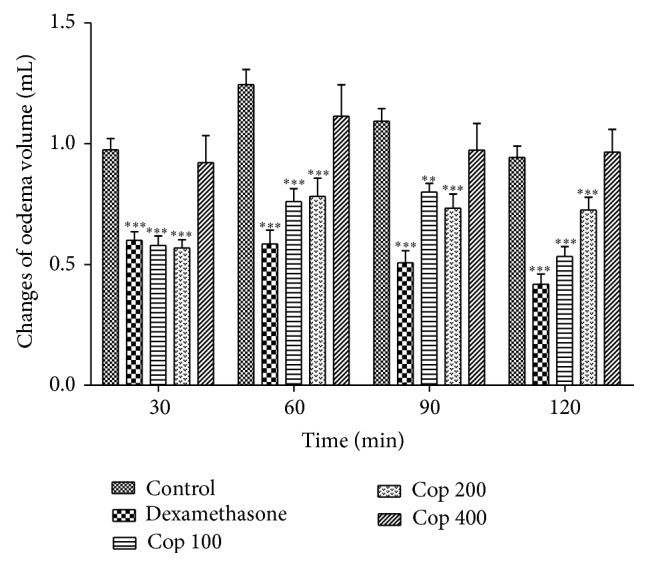
Effect of* C. langsdorffii* extract (Cop) and indomethacin treatments on dextran-induced rat paw oedema. Results are shown as mean oedema volume ± SEM. Time presented in minutes at 30, 60, 90, and 120 minutes after dextran treatment. Statistical difference in comparison with the control group (^∗∗^ *P* < 0.01 and ^∗∗∗^ *P* < 0.001).

In carrageenan-induced oedema Cop was able to reduce the oedema formation at 2 to 4 h for the three tested doses (*P* < 0.001) with a maximal inhibition of 52.3% at 4 h but at 5 h only the dose of 400 mg/kg displayed activity (*P* < 0.01) (Figure 4). Using dextran as the edematogenic agent, the doses of 100 and 200 mg/kg of Cop significantly reduced the oedema at all times with a maximal inhibition of 43.6% (Figure 5).

### 3.5. Evaluation of Peritoneal Cells Migration

LPS was able to significantly induce the migration of cells and increase the number of neutrophils in relation to the number of mononuclear cells into abdominal cavity, but only the dexamethasone was able to significantly inhibit the induction caused by LPS (data not shown).

### 3.6. In Vitro Macrophages Evaluation

The different concentrations of Cop alone (30, 60, and 120 *μ*g/mL) or associated with LPS did not affect the cell viability in comparison to nonstimulated cells (data not shown). Based on these results in MTT assay, the concentrations used in the following experiments were 30, 60, and 120 *μ*g/mL of Cop.

**Figure 6:**
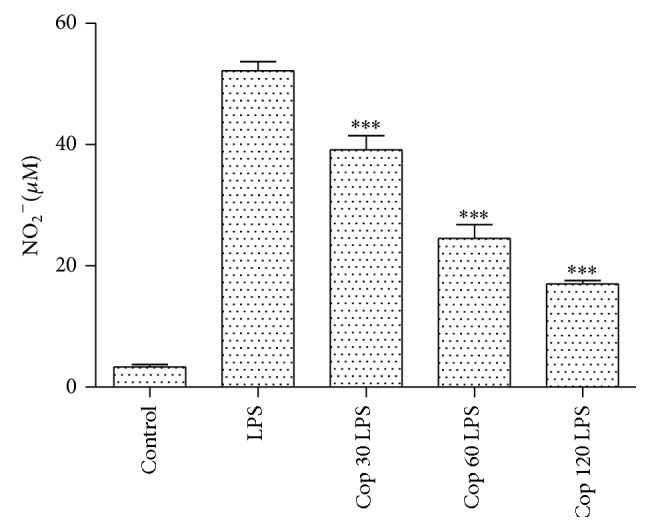
Cop effects on nitric oxide production in the presence of LPS. The supernatants were collected 24 h after adherent cells had been prestimulated with LPS (0.4 *μ*g/mL). Cop (30, 60, and 120 *μ*g/mL) was added 2 h after LPS stimulation. The amount of NO present in the supernatant was determined by Griess reaction. The results are expressed as mean ± SEM of two independent experiments. ^∗∗∗^ *P* < 0.001 compared to LPS alone.

In the present study Cop at tested concentrations significantly reduced the LPS-induced NO production (*P* < 0.001) (Figure 6), and it did not have any effect on NO release without LPS-induction (data not shown). However, Cop at 30, 60, and 120 *μ*g/mL neither stimulated nor displayed a statistically significant reduction of the TNF-*α*, IL-6, and IL-10 production induced by LPS (data not shown).

## 4. Discussion

The inflammatory process involves enzyme activation, mediator release, extravasation of fluids, cell migration, and tissue breakdown [18, 19]. Therefore, it is important to use different experimental models to evaluate the analgesic and anti-inflammatory activities of medicinal plants to further investigate their mechanism of action.

Cop, quercitrin, and afzelin were neither active nor toxic in depressing the central nervous system. Then, the writhing test induced by acetic acid as a triage of nonnarcotic and narcotic analgesics was performed [9]. It consists of intraperitoneal injection of acetic acid as noxious agent. Tissue damage leads to the release of inflammatory mediators, and the activity observed in writhing can be associated with local anesthetics, analgesic, and anti-inflammatory activities [20]. However, to verify whether the observed reduction was caused by analgesic or anti-inflammatory activities, the formalin test was performed.

Quercetin and kaempferol, the aglycones of quercitrin and afzelin, respectively, inhibit the release of IL-6 and IL-8, TNF-*α*, histamine, and tryptase, showing anti-inflammatory activity [21]. However, in the present study, quercitrin and afzelin were inactive, and they differ from quercetin and kaempferol by the presence of sugar moieties linked to the oxygen at carbon 3 of C ring. The inactivity of glycosylated quercetin and kaempferol was also observed by Brown and Dietrich [22]. They reported that quercetin and kaempferol are mutagenic in the microsome assay in* Salmonella*/mammalian TA98. However, when glycosylated (quercetin-3-*O*-rhamnoside and kaempferol-3-*O*-galactoside-rhamnoside-7-*O*-rhamnoside) they did not display mutagenic activity. Therefore, considering the inactivity of quercitrin and afzelin the subsequent tests for these compounds were not undertaken.

The administration of Cop inhibited only the second phase of formalin test. Formalin test is used to evaluate how an animal responds to moderate and continuous pain caused by tissue injury. The first phase (0–5 min) has been thought to result from direct activation of nociceptive afferent fibers indicating that Cop did not display analgesic activity, which was confirmed by the absence of activity in the hot plate test. The second phase (15–30 min) is an inflammatory peripheral process [10], which indicates that Cop displays a peripheral anti-inflammatory activity.

Carrageenan-induced paw oedema involves many mediators, which induce inflammatory reaction in two different phases. The initial phase, which occurs 1 h after the injection of the carrageenan, has been attributed mainly to the action of mediators such as histamine, serotonin, and bradykinin on vascular permeability. On the late phase in carrageenan-induced paw oedema, Cop efficiently suppressed the oedema suggesting the inhibitory effect on different mediators, including overproduction of prostaglandins, cyclooxygenase products, and NO [23, 24]. Dextran is characterized by fluid extravasation and oedema formation in rats. van Wauwe and Goossens [25] showed that dextran-provoked inflammation was suppressed by serotonin antagonists and lipoxygenase inhibitors. Therefore, the results showed that Cop inhibit dextran-induced plasma extravasation. On the other hand, a selective antihistaminic that lacks antiserotonin activity failed to block dextran-induced vascular permeability changes, ruling out a participating role for histamine in this type of inflammatory reaction [26]. Therefore, the weak activity observed for Cop on carrageenan-induced paw oedema at the 1 h could be attributed to the participation of histamine in carrageenan-induced paw oedema.

In the present study Cop at tested concentrations significantly reduced the LPS-induced NO production and they did not have an effect on NO release without LPS-induction. NO is an effector molecule synthesized from the amino acid l-arginine and can be released in response to receptor and physical stimulation, as well as after activation of different cells as macrophages. When NO is produced in small amounts it shows regulatory functions and contributes to maintaining the integrity and function of the membrane, but in large amounts it displays cytotoxic activity against microorganisms and effect on the inflammatory response as an important vasodilator and antiaggregating [27]. Thus, the obtained results show that NO is an important pathway of edema inhibition by Cop.

LPS is a membrane component of bacteria that can induce responses such as fever. Many of these responses can be attributed to LPS-mediated cytokine production [28]. Considering this and the results on NO release, we investigated Cop ability to stimulate macrophage production of pro- (TNF-*α*, IL-6) and anti-inflammatory (IL-10) cytokines. However, Cop did not stimulate or display a statistically significant reduction of the TNF-*α*, IL-6, and IL-10 production induced by LPS, indicating that this probably is not one of the action paths.

In summary, we showed that the oral treatment of animals with Cop reduced the inflammation induced by chemical agents, whereas quercitrin and afzelin were not involved. Furthermore, Cop is noncytotoxic at the concentrations analyzed by MTT assay and it was noncytotoxic on Swiss mouse peripheral blood [4]. Therefore, Cop did not display central analgesic activity. Also, the antiedematogenic activity of Cop might be associated with the regulation of NO release and probably the regulation of mediators, such as serotonin, on vascular permeability. However, this activity was not associated with cell influx, TNF-*α*, IL-6, and IL-10 regulation.
